# Characteristics of Mitochondrial Genomes and Phylogenetic Analysis of Three Species of Littorinimorpha Snails

**DOI:** 10.3390/ani16142248

**Published:** 2026-07-20

**Authors:** Xumin Wang, Minglei Li, Xiaofei Lu, Chengen Tu, Fuyang He, Sutao Li, Dongyue Gu, Tianyi Liu, Pengyu Qu, Zhikai Xing, Shuang Wang, Lijun Wang, Jiangyong Qu

**Affiliations:** College of Life Science, Yantai University, Yantai 264005, China

**Keywords:** mitogenome, phylogeny, Littorinimorpha, *D. vittatus*, *T. chinensis*, *P. glaucum*

## Abstract

**Simple Summary:**

The order Littorinimorpha includes a vast diversity of snails, but the evolutionary relationships among its major groups remain debated. To address this, we decoded the complete mitochondrial genomes of three snail species: *Doxander vittatus*, *Tonna chinensis*, and *Phalium glaucum*. Our analysis revealed that these genomes are highly conserved in structure. By comparing them with many other species, we reconstructed a detailed family tree. This tree confirms that Tonnoidea forms a distinct evolutionary branch, but surprisingly shows that Stromboidea does not, indicating that its current classification needs revision. Our study provides crucial genetic data and new insights into the evolutionary history of these ecologically important snails.

**Abstract:**

After several revisions, Littorinimorpha has been classified as an order-level taxonomic unit under the subclass Heterobranchia of the class Gastropoda, but its suborder classification system remains controversial. In this study, high-throughput sequencing technology was used to obtain the complete mitochondrial genomes of three species from Stromboidea and Tonnoidea: *Doxander vittatus*, *Tonna chinensis*, and *Phalium glaucum*. Genome analysis revealed that the genomes of these three species are highly consistent in structure. They all contain 13 protein-coding genes (PCGs), 22 tRNA genes, and 2 rRNA genes and lack a typical long non-coding control region. The phylogenetic tree constructed based on PCGs shows that Tonnoidea is a monophyletic group within Littorinimorpha, while Stromboidea is a paraphyletic group. Vermetoidea was identified as a separate evolutionary branch under this order. The present study clarifies the mitochondrial genomic characters of three species, *D. vittatus*, *T. chinensis* and *P. glaucum* and their positions in Littorinimorpha, which provides an important basis for evolutionary and phylogenetic studies of this order.

## 1. Introduction

As one of the most ecologically diverse groups within the Gastropoda, Littorinimorpha plays multiple roles in intertidal ecosystems [1]. Due to their high sensitivity to environmental changes, they are not only a key food source for higher trophic level organisms but also important indicator species for monitoring purposes [2,3]. In addition, Littorinimorpha has significant application value in the fields of aquaculture resource development and the extraction of bioactive substances. Modern taxonomic studies indicate that Littorinimorpha comprises 16 superfamilies and 73 families, exhibiting a high degree of species differentiation [4,5]. However, there are still many discrepancies and controversies regarding the phylogenetic relationships among the major groups within this order. For example, early classification systems (such as the WoRMS database) classified the Xenophoridae family as a separate superfamily, Xenophoroidea [6]. However, the latest molecular systematics research has classified Xenophoridae as belonging to Stromboidea after conducting comparative analyses of the mitochondrial genomes of Stromboidea and its closely related groups (including Calyptraeoidea, Cypraeoidea, Tonnoidea, etc.). This not only resolves long-standing taxonomic disputes, but also corroborates the evolutionary hypothesis based on morphological characteristics proposed by Simone et al. [6,7,8,9]. In addition, there is controversy between the traditional morphological classification of Vermetidae and modern molecular systematics data [10]. Phylogenetic analysis indicates that Vermetidae forms a sister group with other taxa within Caenogastropoda, suggesting that Vermetidae and other members of Caenogastropoda share a common ancestor and may have undergone rapid morphological evolution. However, this conclusion differs significantly from those based on traditional characteristics such as shell morphology and mollusk anatomy [7,10]. This contradiction between “molecular” and “morphological” evidence may reflect the unique evolutionary history of Vermetidae, which warrants further study. Furthermore, many species of Littorinimorpha (especially those that are rare or difficult to sample) lack complete genomic information, limiting our ability to perform high-resolution phylogenetic analyses using whole-genome data [4,11,12]. Under these conditions, traditional methods based on genetic distance may not be sufficient to accurately distinguish between these species. To address this issue, researchers are working to adopt more advanced technical methods (such as whole-genome sequencing, multi-gene joint analysis, Bayesian inference, etc.) and comprehensively consider factors such as incomplete gene sequence ordering to more accurately reconstruct phylogenetic relationships. Advances in these technologies are expected to bring new breakthroughs to the systematic classification research of Littorinimorpha.

Due to their small size, compact structure, low recombination rate, and multiple copies within cells, mitochondrial genomes have become important tools in molecular biology research. They have also demonstrated broad application value in fields such as population genetics, molecular evolution, comparative genomics, and evolutionary genomics [13,14]. The mitochondrial genome contains 13 typical functional genes (such as COI, ND series, Cytb, etc.), which have low recombination rates and are highly conserved, providing reliable molecular markers for phylogenetic reconstruction and population genetic analysis [15]. By jointly analyzing these genes, it is possible to construct a more comprehensive and accurate phylogenetic relationship [16], thereby deeply analyzing the evolutionary history and adaptive mechanisms of species.

*Doxander vittatus*, *Tonna chinensis*, and *Phalium glaucum* are three species with typical ecological characteristics in Littorinimorpha, mainly inhabiting the intertidal zone. They are sensitive to environmental changes but have strong adaptability [17]. As a typical intertidal resident, *D. vittatus* exhibits shell structure characteristics adapted to tidal rhythms [18]; *T. chinensis* is a large predatory snail species. With its unique hunting strategy, it plays an extremely important role in the benthic ecosystem [19]; *P. glaucum* is widely distributed in tropical and subtropical waters. Because its population dynamics are closely related to the health of coral reefs, it has become an important indicator for assessing the health of coral reef ecosystems [19]. Therefore, studying the genetic structure of these species is crucial to maintaining ecological balance and protecting biodiversity [20,21].

In this study, high-quality mitochondrial genome data for *D. vittatus*, *T. chinensis*, and *P. glaucum* were generated using third-generation sequencing technology, and the assembly and annotation of these genomes were completed using bioinformatics tools. Based on this, a phylogenetic tree of the relevant species was reconstructed and the divergence times of the various clades of Littorinimorpha were estimated. The evolutionary history and taxonomic relationships of Littorinimorpha were systematically investigated through phylogenetic analysis, clarifying its evolutionary position within the mollusks and deepening the understanding of mollusk biology. Additionally, these results provide valuable data resources for evolutionary biology research.

## 2. Materials and Methods

### 2.1. Sample Collection and DNA Extraction

To perform complete mitochondrial genome sequencing, live samples of *D. vittatus*, *T. chinensis*, and *P. glaucum* were collected from Sanya City, Hainan Province, China (geographic coordinates: 18°20′52.52″ N, 109°53′37.63″ E). Following collection, muscle tissue samples were promptly extracted from each specimen under stringent aseptic conditions, swiftly transferred to nitrogen-cooled cryogenic vials, and preserved at a temperature of −80 °C within an ultra-low temperature freezer. The genomic DNA was extracted using the DNeasy Blood & Tissue Kit (Qiagen, Beijing, China). Subsequently, deoxyribonucleic acid (DNA) was fragmented into 500 base pair segments utilising the Covaris M220 system (Covaris, Woburn, MA, USA), and short-insert libraries were constructed employing the TruSeq™ Nano DNA Sample Prep Kit (Illumina, San Diego, CA, USA). The final libraries were subjected to sequencing using the Illumina NovaSeq 6000 platform (Illumina, San Diego, CA, USA) at Shanghai Biozeron Biotechnology Co., Ltd (Shanghai, China), with dual-end 150 bp sequencing.

### 2.2. High-Throughput Sequencing and Assembly

Sequencing of the three libraries was performed, yielding raw data with an average coverage depth of 300×. The raw data were subjected to strict quality control processing using Trimmomatic v0.39 (http://www.usadellab.org/cms/?page=trimmomatic; accessed on 11 June 2025): Illumina universal adapters and species-specific contamination sequences were removed; non-AGCT bases at the 5′ end were trimmed; bases with Phred quality scores < 20 at the 3′ end were trimmed; reads containing > 10% N bases were removed; and high-quality reads ≥ 75 bp were retained for subsequent analysis. Use the GetOrganelle v1.7.5 software (https://github.com/Kinggerm/GetOrganelle; accessed on 11 June 2025) for mitochondrial genome assembly. The software performs a cyclic search of target reads based on seed sequences, then calls upon SPAdes for genome assembly. In order to ensure the reliable and complete nature of the final assembly results, it was necessary to select sequences with sufficient coverage depth and longer assembly lengths as candidate sequences. In addition, the mitochondrial origin of these sequences was verified by aligning them with the NCBI NT database. Based on the overlapping regions between sequences, multiple alleles were spliced into longer scaffolds. To further optimize the assembly results, a reference genome (OR995294) more closely related to the target species was selected, and BLAST v2.8.1 (https://blast.ncbi.nlm.nih.gov/Blast.cgi; accessed on 11 June 2025) was used to screen for alleles with query coverage exceeding 80%. Based on the comparison results, the alleles were manually aligned and merged, ultimately yielding the complete mitochondrial genome (Table 1).

### 2.3. Annotation and Comparative Genome Analysis

The Galaxy platform (https://usegalaxy.org/; accessed on 11 June 2025) was utilised for the prediction of coding proteins, tRNA, and rRNA genes within the mitochondrial genome. The manual correction of genes with start/stop codons was conducted using the SnapGene Viewer (v8.1.1) and reference mitochondrial genomes (Tables S1–S3) [22]. The secondary structures of all tRNA genes were predicted and visualized using the VARNA applet (v3.9). Furthermore, in order to identify and distinguish between the heavy chain (H chain) and the light chain (L chain) of the mitochondrial genome, the position of each gene was determined on the basis of the known mitochondrial genome structure. It has been established that the heavy chain (HC), which is the subject of this study, contains the majority of protein-coding genes. Examples of these genes include *cytochrome c oxidase subunits 1 and 2* (*COX1* and *COX2*). The HC is abundant in adenine (A) and thymine (T) bases. In contrast, the light chain (L chain) is characterised by a reduced number of protein-coding genes (e.g., ND6, CytB, etc.) and a relative abundance of guanine (G) and cytosine (C) bases [13,23]. The Organelle Genome Drawing (OGDRAW) tool (https://chlorobox.mpimp-golm.mpg.de/OGDraw.html; accessed on 13 June 2025) was used to perform physical mapping of the three mitochondrial genomes (Table 2) [24]. Relative synonymy codon usage (RSCU) was determined through the utilisation of the CUSP tool within the EMBL-EBI bioinformatics suite [25]. In the event that all synonymous code points for a given amino acid are utilised equally (i.e., no usage preference), the RSCU value for each codon is 1. In the event of a codon being employed at a frequency that exceeds its theoretical expected value (i.e., a usage preference is in evidence), its RSCU value will exceed 1. Conversely, in instances where the frequency of usage of a codon falls below the theoretical expected value, its RSCU value is bound to be less than 1. By analyzing RSCU values, differences in codon usage patterns between species were identified, and potential mechanisms regulating mitochondrial gene expression were inferred. The following formulae are employed in order to calculate AT bias and GC bias:

(1) $$AT\;skew=\left(A-T\right)∕\left(A+T\right)$$

(2) $$GC\;skew=\left(G-C\right)∕\left(G+C\right)$$

### 2.4. Phylogenetic Analysis

This study integrated 99 mitochondrial genomes of Littorinimorpha from GenBank (NCBI) (see Supplementary Table S4 for details, including accession numbers, references, and publication status) and combined them with three newly sequenced genomes to conduct a phylogenetic analysis (Table 3). The study concentrated on 13 mitochondrial protein-coding genes (mtPCGs). The nucleotide sequences of these 13 PCGs were extracted from each complete mitogenome using the batch extraction function in PhyloSuite v1.2.2 [26]. We then utilised Bayesian inference (BI) and maximum likelihood (ML) methodologies to construct the phylogenetic tree. To optimize the analysis results, *Lunella correensis* (NC_081576) and *Haliotis rubra* (NC_005940) were selected as outgroups. This selection strategy not only ensured clear differentiation between the outgroup and the study species but also effectively revealed the phylogenetic relationships within the family Haliotidae, significantly reducing potential phylogenetic tree biases caused by inappropriate outgroup selection, thereby establishing a reliable reference framework for phylogenetic assessment.

In the sequence alignment phase, MAFFT (V 7.313) [27] (https://mafft.cbrc.jp/alignment/software/; accessed on 15 June 2025) was used to perform precise alignments of the relevant sequences. Subsequently, the aligned mitochondrial protein-coding gene sequences were concatenated into a matrix, which provided a robust data foundation for subsequent phylogenetic analysis. The establishment of the optimal partition model was informed by the Akaike Information Criterion (AIC). This was achieved through the utilisation of PartitionFinder2 (V 2.21) (http://www.robertlanfear.com/partitionfinder/; accessed on 15 June 2025) [28]. The best-fitting model for nucleotide substitutions was determined using IQ-TREE Model Selection and the +R option (FreeRate model) [29]. Based on this, maximum likelihood (ML) guided analysis was conducted using RAxML v8.2.12 (https://cme.h-its.org/exelixis/web/software/raxml/index.html; accessed on 15 June 2025) to further reconstruct the phylogenetic tree [30], and 1000 guided analyses were used to accurately estimate the reliability of each node. A phylogenetic tree was finally constructed on the basis of the mitochondrial genomes of the three target species and 99 Littorinimorpha species (Table 3). The relative positions of each branch are clearly labelled.

### 2.5. Estimation of Divergence Time

To accurately estimate evolutionary divergences amongst the various Littorinimorpha clades, a meticulous investigation was conducted on a selection of 13 protein-coding genes (PCGs) at the nucleotide level. The estimation of species divergence times was achieved through the implementation of the uncorrelated log-normal relaxed clock model in BEAST v1.10.4 (https://beast.community/2018-11-14_BEAST_v1.10.4_released.html; accessed on 15 June 2025) [31,32]. This model enables the examination of evolutionary rates as a function of their independence across disparate evolutionary branches, thus obviating the necessity for adjacent branches to exhibit autocorrelated rates. The Yule process was employed as the predecessor to the tree model, and the optimal evolutionary model (GTR + I + G) was selected for analysis. The Bayesian approach to inference was implemented utilising MrBayes 3.2.7 (http://nbisweden.github.io/MrBayes/; accessed on 15 June 2025). In the course of the Bayesian Markov Chain Monte Carlo (MCMC) analysis, it was established that two independent runs were initiated, with each comprising four parallel chains. The total number of generations that were iterated was 750,000,000, with the sampling interval set at 1000 generations [33]. In order to guarantee the reliability of the analysis results, the initial 75,000 generations (representing the initial 10%) of samples were set aside for the purpose of burn-in. A phylogenetic tree was generated based on the posterior probability values, which was then used to assess the support levels of each node. Meanwhile, the convergence of the chain was confirmed with the help of Tracer v1.7.2 (https://github.com/beast-dev/tracer/releases/tag/v1.7.2; accessed on 17 June 2025) to ensure that the Effective Sample Size (ESS) for all the parameters was more than 200. The generation of the maximum branch credibility tree was finally achieved using TreeAnnotatorv1.10.4 (https://www.beast2.org/treeannotator/; accessed on 17 June 2025) to produce the maximum branch confidence tree [28]. The key node time estimation integrates log-normality constraints for five fossil calibration points: (1) *Lunella correensis* and *Haliotis rubra* divergence times ranged from 220.0 to 398.9 million years ago (Mya) [34]. (2) *Maackia herderiana* and *Potamopyrgus estuarinus* diverged times ranged from 126.8 to 140.0 Mya [35,36]. (3) *Tricula hortensis* and *Oncomelania hupensis robertsoni* divergence times ranged from 20.2 to 65.8 Mya [35,37,38]. (4) *Littoraria melanostoma* and *Notocochlis gualtieriana* divergence times ranged from 119.1 to 122.0 Mya [39]. (5) *Bufonaria rana* and *Bursa rhodostoma* divergence times ranged from 34.9 to 35.5 Mya [40]. In order to guarantee congruence between the upper 95% posterior density interval and the upper soft bound, the mean of the lognormal distribution (μ) and its standard deviation (σ) were modified using the fossil record as a lower limit constraint. The final constructed timing tree was visualised and presented by FigTree v1.4.4 (http://tree.bio.ed.ac.uk/software/figtree/; accessed on 15 June 2025).

## 3. Results

### 3.1. Genome Features

**Figure 1:**
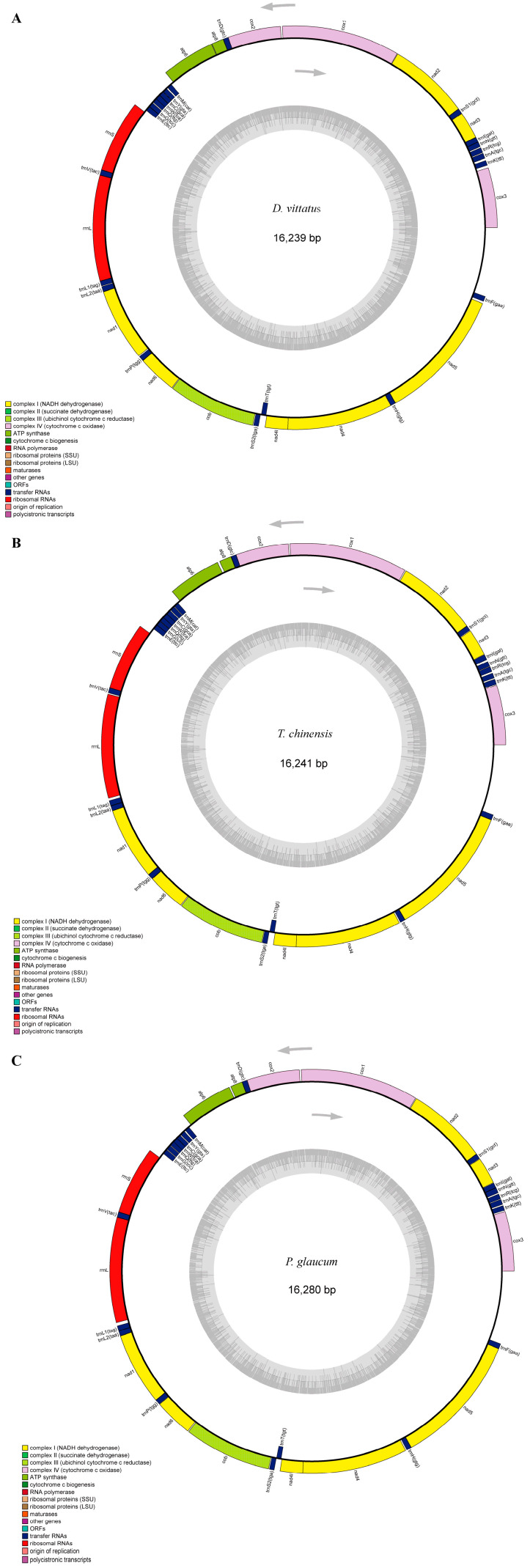
Gene map of the complete mitochondrial genomes of *D. vittatus* (**A**), *T. chinensis* (**B**) and *P. glaucum* (**C**). The regions exhibiting colouration are indicative of protein-coding and rRNA genes, whilst dark blue regions are associated with tRNA genes. The outer circle is indicative of the positive (+) strand, whilst the inner circle denotes the negative (−) strand. Arrows indicate the direction of gene transcription.

The mitochondrial complete genome lengths of *D. vittatus*, *T. chinensis*, and *P. glaucum* are 16,239 bp, 16,241 bp, and 16,280 bp, respectively, with average GC contents of 32.83%, 28.48%, and 29.22%, respectively. The complete mitochondrial genomes of the three species collectively encode 37 genes, including 13 PCGs, 22 transfer RNA (tRNA) genes, and 2 ribosomal RNA (rRNA) genes (Figure 1). Their genomic structures are relatively stable and highly conserved. Notably, a typical, long non-coding control region was not identified in any of the three mitogenomes. The sequences between *trnF* and *cox3* are very short (ranging from 6 to 33 bp) and lack conserved sequence blocks characteristic of a control region, which is a feature observed in some other gastropod mitogenomes [7,41]. The length of tRNA has been determined to range from 63 base pairs (bp) to 72 bp, thus confirming its consistency with the typical characteristics of gastropods. The total lengths of the PCGs are 11,262 bp, 11,250 bp, and 11,247 bp, respectively, accounting for 69.35%, 69.27%, and 69.08% of the total mitochondrial gene count (Table 4). The 13 PCGs include 3 cytochrome cofactor subunits (*cox1-3*), 7 NADH dehydrogenase subunits (*nad1-6* and *nad4l*), 1 cytochrome b gene (cob), and 2 ATP synthase subunits (*atp6* and *atp8*), with *nad5* being the longest and *atp8* the shortest.

The distribution of heavy chain and light chain genes within the mitochondrial genomes of the three species is characterised by a high degree of consistency. Specifically, the majority of these genes are located in the heavy chain; in the light chain, there are only 8 tRNA genes: *trnM*, *trnY*, *trnC*, *trnW*, *trnQ*, *trnG*, *trnE*, and *trnT* (Figure 1). Among the 13 PCGs, *nad5* is the longest gene, with a length of 1728 bp in *D. vittatus* and 1722 bp in *T. chinensis* and *P. glaucum*; atp8 is the shortest gene, with a length of only 159 bp. *rrnS* ranges from 887 to 847 bp in length, while *rrnL* ranges from 1352 to 1372 bp in length (Table 5). These two genes are adjacent in the genome, both located between *trnE* and *trnL1*, and separated by the *trnV* gene. The distribution and arrangement pattern is consistent across the three species, further emphasising the high level of conservation of the mitochondrial genome in question.

**Figure 2:**
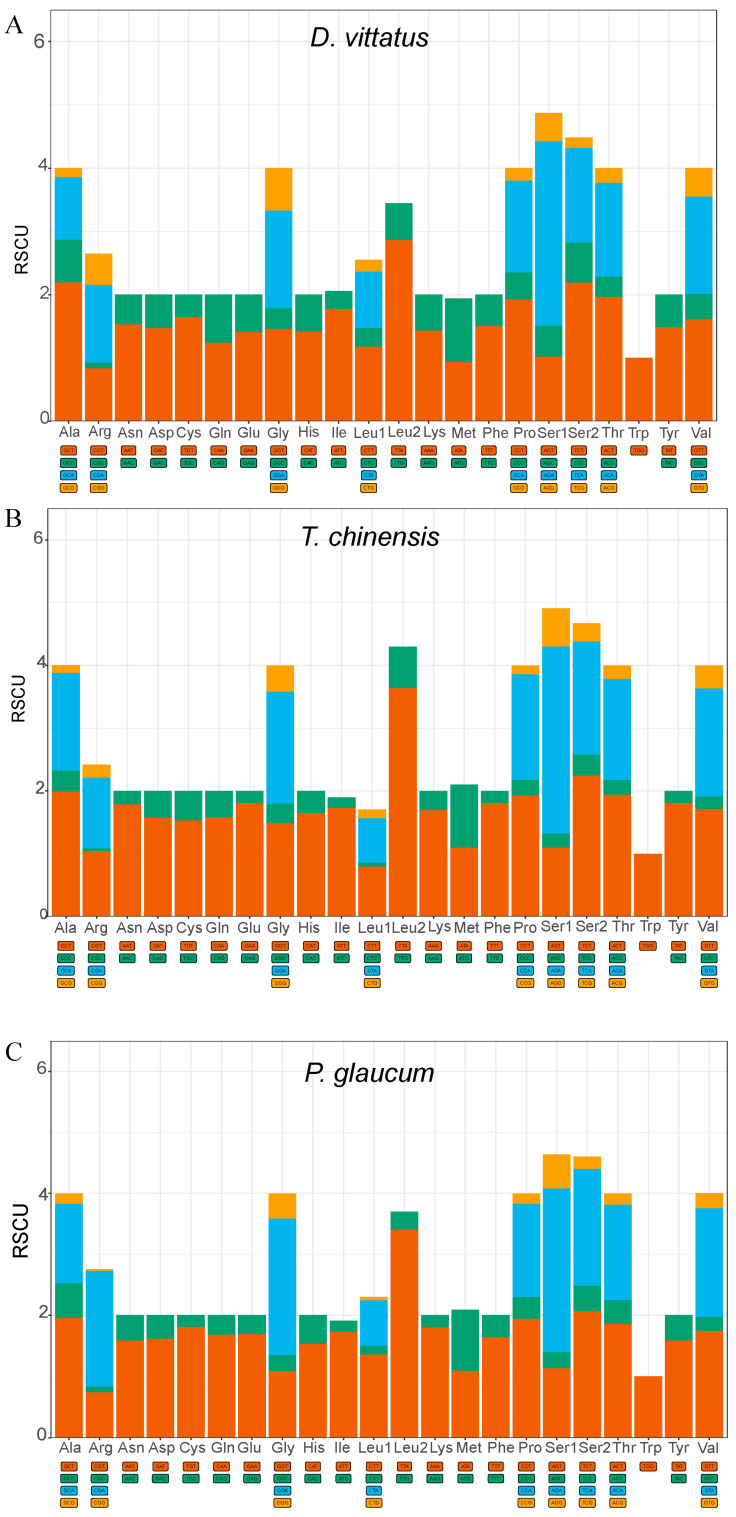
Relative synonymous codon usage (RSCU) in the mitochondrial genomes of three species. (**A**): *D. vittatus*; (**B**): *T. chinensis*; (**C**): *P. glaucum*. The horizontal coordinates indicate the amino acids and their corresponding codons, and the vertical coordinates indicate the RSCU values. The height of each bar indicates the relative synonymous codon usage (RSCU) of the corresponding amino acid, and different colors are used to distinguish different synonymous codons.

**Figure 3:**
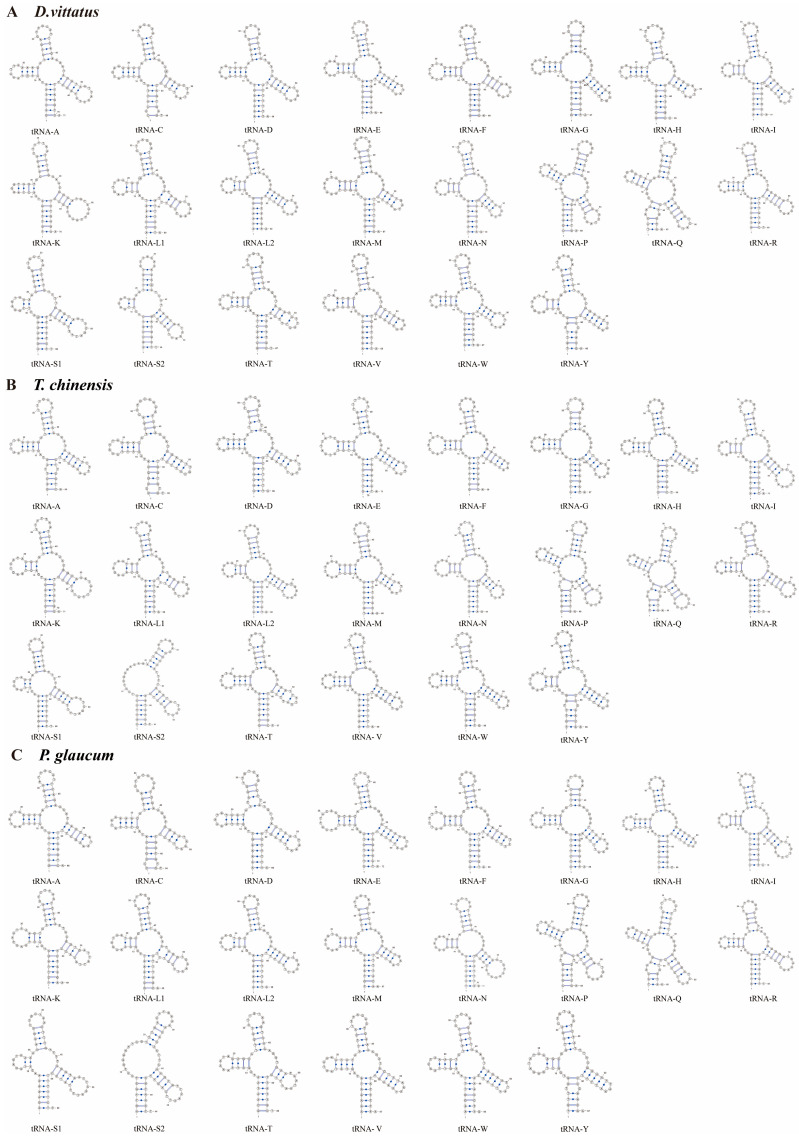
The secondary structure of tRNA in the mitochondrial genomes of *D. vittatus* (**A**), *T. chinensis* (**B**), and *P. glaucum* (**C**).

In the three mitochondrial genomes, the AT and CG contents of genes encoding proteins, tRNA, and rRNA were consistent with the base distribution of the entire genome (Table S5). Among the 13 PCGs, most use ATG as the start codon, but some PCGs use ATT or ATA as the start codon. All PCGs use TAA or TAG as the stop codon. The relative synonymous codon usage RSCU values of the 13 PCGs indicate that the two most common codons in all three species are AGA and TTA (Figure 2). The most commonly used codons for Ser1, Leu2, Ala, Ser2, and Gly are AGA, TTA, GCT, TCT, and GGA, respectively (Table S6). Prediction of the secondary structure for each tRNA revealed that, except for tRNA-S2 in *T. chinensis* and *P. glaucum* lacking the D-loop and D-arm, all other tRNAs exhibit a typical cloverleaf structure (Figure 3). Additionally, among the 22 tRNA structures, most G-T mismatches occur on the amino acid acceptor arm.

### 3.2. Phylogenetic Relationships

**Figure 4:**
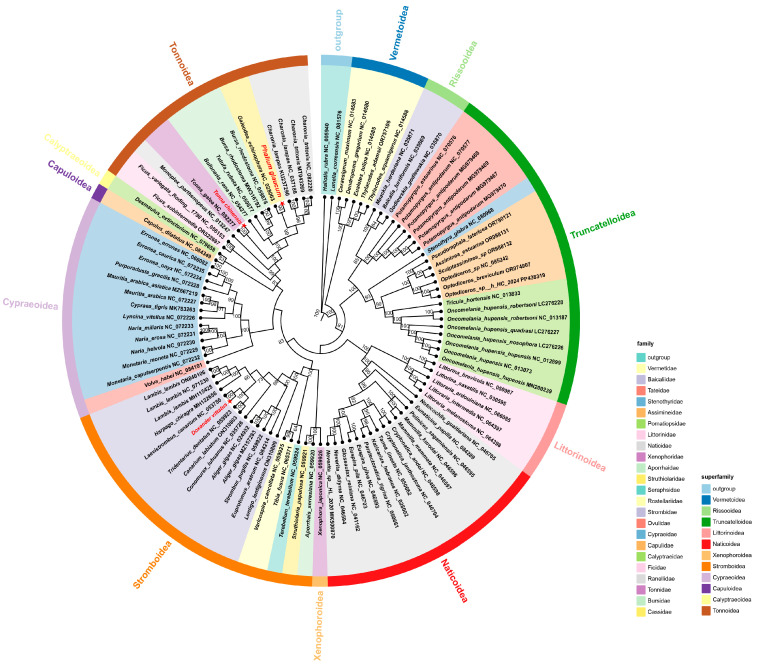
Phylogenetic tree of Littorinimorpha based on nucleotide sequences of 13 mitochondrial PCGs. Bootstrap support values from maximum likelihood analysis are shown at the nodes. Red stars highlight the three species in this study.

The phylogenetic tree constructed based on mitochondrial genome data from three species showed that most node bootstrap values reached 100% (using the maximum likelihood method) and posterior probabilities reached 1.00 (using Bayesian inference), indicating that the constructed phylogenetic tree has extremely high reliability (Figure 4 and Figure S1). A phylogenetic analysis was conducted on 102 species of Littorinimorpha, covering 23 genera and 11 families (Figure 4). Among these species, *D. vittatus* and *Laevistrombus canarium*, as well as *Tridentarius dentatus* and *Canarium labiatum*, all belong to Stromboidea. In addition, Stromboidea is more closely related to Xenophoroidea, and together they form a unique evolutionary branch. Meanwhile, *T. chinensis* and *P. glaucum* belong to the family Tonnoidea, where *T. chinensis* was most closely related to *Tonna galea*, while *P. glaucum* shares the same evolutionary branch as *Galeodea echinophora*. Notably, the family Tonnoidea, which includes six families, exhibits monophyletic groups and shares a common ancestor with Capuloidea and Calyptraeoidea, which satisfies the classification of a paraphyletic group.

**Figure 5:**
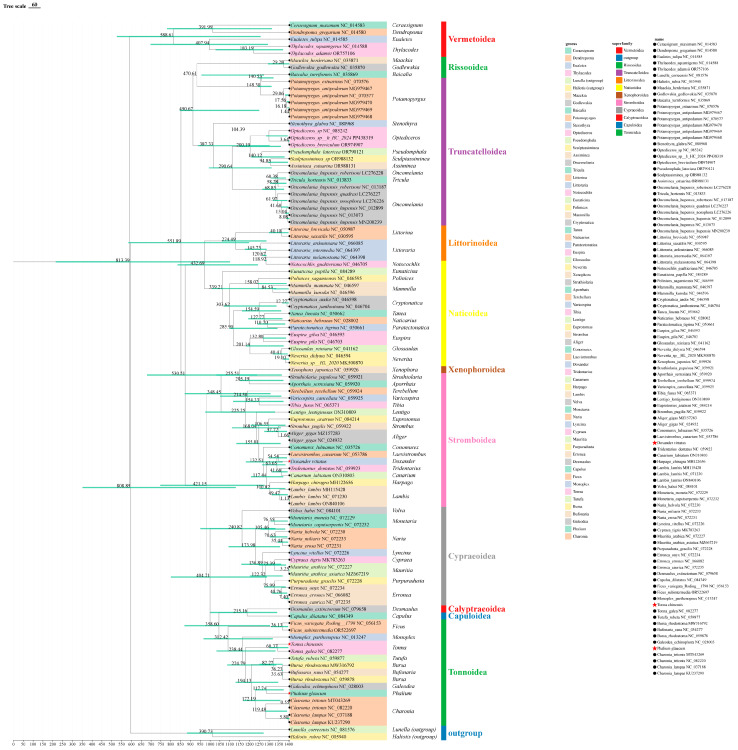
Divergence time estimation for Littorinimorpha inferred with BEAST based on 13 PCGs. The 95% Highest Posterior Density (HPD) is reported using green bars.

Species divergence times were estimated utilizing the BEAST software. The time-calibrated phylogenies demonstrated that *D. vittatus* and *L. canarium* diverged 54.54 Mya, while *T. chinensis* and *T. galea* diverged 60.37 Mya. Both divergence events occurred during the Paleogene period of the Cenozoic Era. *P. glaucum* and *G. echinophora* had a differentiation time of 117.74 Mya and belonged to the Mesozoic Cretaceous (Figure 5).

## 4. Discussion

By exhaustively sequencing and analyzing the mitochondrial genomes of three species, *D. vittatus*, *T. chinensis* and *P. glaucum*, we have gained important insights into these species and their evolutionary relationships. The complete mitochondrial genomes of these three species were 16,239 bp, 16,241 bp and 16,280 bp in length, with an average GC content of 32.83%, 28.48% and 29.22%, respectively, which is consistent with the typical structural features of the mitochondrial genomes of species of the Littorinimorpha [20]. Despite minor differences in total genome length, the PCGs of the three species show high consistency in total length, at 11,262 bp, 11,250 bp, and 11,247 bp, respectively. In addition, these genes exhibit significant differences in base composition, particularly in GC content, the underlying causes of which are multifaceted and may include both neutral and selective processes [42,43,44]. For instance, the variation in GC content (e.g., 32.83% in *D. vittatus* vs. 28.48% and 29.22% in *T. chinensis* and *P. glaucum*, respectively) could potentially be associated with their distinct habitats and life history traits, although it is also strongly influenced by lineage-specific mutation rates and deep phylogenetic signals [40]. Further studies integrating population-level data and functional assays would be needed to elucidate the relative contributions of these evolutionary forces. In all three species, the length of tRNA genes ranges from 63 bp to 72 bp, highlighting their importance in post-transcriptional modification processes [22]. Notably, among the 13 PCGs, the longest is *nad5*, while the shortest is *atp8*. This length variation not only reflects the functional requirements of different genes but may also influence translation efficiency and protein stability [45,46]. An intriguing finding beyond the general genomic features was the structural divergence in the secondary structure of the tRNA-S2 (*trnS2*) molecule. Research indicates that the molecules of *T. chinensis* and *P. glaucum* both lack the D arm and D ring structures, whereas *D. vittatus* exhibits a complete cloverleaf structure. As a crucial component involved in enzyme recognition and structural stability maintenance [47], the absence of the D arm highlights the plasticity of mitochondrial evolution [48]. This structural variant shared by the two tonnoidean species (*T. chinensis* and *P. glaucum*) but not by the stromboid (*D. vittatus*) may represent a synapomorphy for Tonnoidea or a convergent adaptation, warranting further investigation into its functional consequences.

The phylogenetic tree constructed in this study supports the three main branches previously recognized for Littorinimorpha: (1) Stromboidea, Tonnoidea, Littorinoidea, and Naticoidea; (2) Rissooidea and Truncatelloidea; (3) Vermetoidea. Unlike earlier studies, it further extends the scope of the first branch by adding several superfamilies. In addition to the four previously reported superfamilies [7], it supports that Xenophoroidea, Cypraeoidea, Capuloidea, and Calyptraeoidea also belong to this branch. Moreover, it suggests a close relationship between Stromboidea and Xenophoroidea, although the two superfamilies exhibit varied morphological features [35,49]. This finding suggests that Stromboidea is not a monophyletic group. This breakthrough discovery greatly challenges our traditional understanding of the phylogenetic relationships within the Littorinimorpha system, highlighting the need to reevaluate the relationships between species within Stromboidea [50]. The above results indicate that it is necessary to reevaluate the current classification framework, especially those classifications based on morphological characteristics rather than molecular data [35,36]. This study proposes the use of molecular data, such as mitochondrial genomic information, to redefine the taxonomic units of Stromboidea and Truncatelloidea. For example, species such as *Struthiolaria papulosa* and *Aporrhais serresiana*, which do not belong to a monophyletic group [51,52], should be considered for exclusion from the Stromboidea superfamily or reclassification into more appropriate taxonomic units. Members of Littorinimorpha are characterized by their delicate shell morphology, unique locomotion patterns, and typically large, colorful eyes, and possess significant commercial value (e.g., for shell trade and fisheries) [11,23]. With the accumulation of molecular data, the traditional morphological classification system is facing major revisions. This study highlights the necessity of integrating mitochondrial genomic data to construct the taxonomic classification of Littorinimorpha, particularly the re-evaluation of the Stromboidea and Truncatelloidea. This will provide a novel theoretical foundation for future taxonomic research, necessitating increased sampling and combined morphological-molecular analyses.

Environmental change and geographic isolation are established drivers of taxonomic diversification, yet they also complicate phylogenetic reconstruction, particularly when integrated with paleobiogeographic evidence [53,54]. A clear example is found in Vermetidae, where traditional morphology-based classifications conflict with molecular phylogenetic data [7]. Such discordance may reflect a complex evolutionary history, potentially involving processes such as incomplete lineage sorting (ILS)—which can be intensified during rapid radiative adaptations—as well as introgression or convergent evolution [55,56]. The three species studied here—*D. vittatus*, *T. chinensis*, and *P. glaucum*—occupy distinct ecological niches and temporal contexts. *D. vittatus* thrives in highly variable intertidal settings, where fluctuations in tidal regime, temperature, and salinity foster recurrent population isolation [4,18]. *T. chinensis*, by contrast, is associated with unconsolidated sandy or muddy substrates where patchy resource distribution shapes its demographic structure. *P. glaucum* is broadly dispersed across tropical and subtropical coral reefs [57,58]; the structural complexity and high biodiversity of these habitats supply diverse niches, while physical barriers inherent to reef systems facilitate genetic divergence. These ecologically driven patterns of polymorphism enhance our ability to reconstruct phylogenetic relationships, though resolving such complex divergence histories will require expanded genomic datasets and more sophisticated analytical approaches.

The monophyly of Littorinimorpha and its phylogenetic placement within Caenogastropoda have been subjects of ongoing debate [20]. Although traditional morphological evidence supported Littorinimorpha as a monophyletic group [4], the advent of molecular data has substantially refined phylogenetic reconstructions. The present analysis corroborates the monophyly of Littorinimorpha and recovers Vermetoidea as a distinct internal clade, aligning with certain recent studies [59]. Moreover, our findings indicate a sister-group relationship or close affinity between Littorinimorpha and several caenogastropod lineages formerly placed in the polyphyletic assemblage “Mesogastropoda”. The variation in morphological complexity among these groups—from intertidal Littorinimorpha to lineages inhabiting terrestrial environments—appears to be a product of divergent ecological adaptations and evolutionary trajectories, rather than a straightforward transition from simple to complex forms [60,61,62].

## 5. Conclusions

This study employs mitochondrial genomic data to clarify the taxonomic status and phylogenetic positions of *D. vittatus*, *T. chinensis*, and *P. glaucum* within Littorinimorpha. Beyond providing essential genetic resources for these species, our analysis reveals highly conserved genomic architectures alongside nucleotide-level variations, offering insights into both structural constraints and ecological adaptations. Critically, our robust phylogeny supports the monophyly of Tonnoidea but reveals the polyphyly of Stromboidea, highlighting a significant incongruence between molecular data and traditional morphology-based classifications. These findings not only necessitate a re-evaluation of superfamily relationships but also provide a critical molecular foundation for future species identification, conservation efforts, and understanding of evolutionary patterns in this ecologically diverse gastropod order.

## Figures and Tables

**Table 1:** Assembly statistics of the mitochondrial genomes for *T. chinensis*, *D. vittatus*, and *P. glaucum.*

Samples ID	Total Length	N50 Length	N90 Length	Max. Length	Min. Length	GC Content (%)
*T. chinensis*	16,239	16,239	16,239	16,239	16,239	32.83%
*D. vittatus*	16,241	16,241	16,241	16,241	16,241	28.48%
*P. glaucum*	16,280	16,280	16,280	16,280	16,280	29.21%

**Table 2:** Statistics of the protein-coding genes (PCGs) in the mitochondrial genomes of *T. chinensis*, *D. vittatus*, and *P. glaucum.*

Sample ID	Complete Mitogenome Size (bp)	PCG Number	PCG Total Length (bp)	PCG Average Length (bp)	PCG Length/Genome (%)
*T. chinensi*	16,239	13	11,262	866	69.35
*D. vittatus*	16,241	13	11,250	865	69.27
*P. glaucum*	16,280	13	11,247	865	69.08

**Table 3:** List of Littorinimorpha species with complete mitochondrial genomes used in this phylogenetic analysis, sourced from GenBank (NCBI).

Species	Accession	Superfamily	Family	Size
*Lunella correensis*	NC_081576_1	Trochoidea	Turbinidae	17,309 bp
*Haliotis rubra*	NC_005940_1	Haliotoidea	Haliotidae	16,907 bp
*Ceraesignum maximum*	NC_014583_1	Vermetoidea	Vermetidae	15,578 bp
*Dendropoma gregarium*	NC_014580_1	Vermetoidea	Vermetidae	15,641 bp
*Eualetes tulipa*	NC_014585_1	Vermetoidea	Vermetidae	15,078 bp
*Thylacodes squamigerus*	NC_014588_1	Vermetoidea	Vermetidae	15,544 bp
*Thylacodes adamsii*	OR757106_1	Vermetoidea	Vermetidae	14,913 bp
*Maackia herderiana*	NC_035871_1	Rissooidea	Baicaliidae	15,154 bp
*Godlewskia godlewskia*	NC_035870_1	Rissooidea	Baicaliidae	15,224 bp
*Baicalia turriformis*	NC_035869_1	Rissooidea	Baicaliidae	15,127 bp
*Potamopyrgus estuarinus*	NC_070576_1	Truncatelloidea	Tateidae	16,701 bp
*Potamopyrgus antipodarum*	MG979467_1	Truncatelloidea	Tateidae	15,141 bp
	NC_070577_1	Truncatelloidea	Tateidae	16,846 bp
	MG979470_1	Truncatelloidea	Tateidae	15,149 bp
	MG979469_1	Truncatelloidea	Tateidae	15,145 bp
	MG979468_1	Truncatelloidea	Tateidae	15,149 bp
*Stenothyra glabra*	NC_080968_1	Truncatelloidea	Stenothyridae	15,704 bp
*Optediceros* sp.	NC_085242_1	Truncatelloidea	Assimineidae	15,906 bp
*Optediceros* sp. *h HC 2024*	PP438319_1	Truncatelloidea	Assimineidae	15,969 bp
*Optediceros breviculum*	OR974907_1	Truncatelloidea	Assimineidae	15,870 bp
*Pseudomphala latericea*	OR790121_1	Truncatelloidea	Assimineidae	16,635 bp
*Sculptassiminea* sp.	OR988132_1	Truncatelloidea	Assimineidae	16,073 bp
*Assiminea estuarina*	OR988131_1	Truncatelloidea	Assimineidae	15,907 bp
*Tricula hortensis*	NC_013833_1	Truncatelloidea	Pomatiopsidae	15,179 bp
*Oncomelania hupensis robertsoni*	LC276228_1	Truncatelloidea	Pomatiopsidae	15,188 bp
	NC_013187_1	Truncatelloidea	Pomatiopsidae	15,191 bp
*Oncomelania hupensis quadrasi*	LC276227_1	Truncatelloidea	Pomatiopsidae	15,184 bp
*Oncomelania hupensis nosophora*	LC276226_1	Truncatelloidea	Pomatiopsidae	15,182 bp
*Oncomelania hupensis hupensis*	NC_012899_1	Truncatelloidea	Pomatiopsidae	15,186 bp
	NC_013073_1	Truncatelloidea	Pomatiopsidae	15,182 bp
	MN200239_1	Truncatelloidea	Pomatiopsidae	15,850 bp
*Littorina brevicula*	NC_050987_1	Littorinoidea	Littorinidae	16,356 bp
*Littorina saxatilis*	NC_030595_1	Littorinoidea	Littorinidae	16,887 bp
*Littoraria ardouiniana*	NC_066085_1	Littorinoidea	Littorinidae	16,261 bp
*Littoraria intermedia*	NC_064397_1	Littorinoidea	Littorinidae	16,194 bp
*Littoraria melanostoma*	NC_064398_1	Littorinoidea	Littorinidae	16,149 bp
*Notocochlis gualtieriana*	NC_046705_1	Naticoidea	Naticidae	15,176 bp
*Eunaticina papilla*	NC_084289_1	Naticoidea	Naticidae	16,415 bp
*Polinices sagamiensis*	NC_046595_1	Naticoidea	Naticidae	15,383 bp
*Mammilla mammata*	NC_046597_1	Naticoidea	Naticidae	15,319 bp
*Mammilla kurodai*	NC_046596_1	Naticoidea	Naticidae	15,309 bp
*Cryptonatica andoi*	NC_046598_1	Naticoidea	Naticidae	15,302 bp
*Cryptonatica janthostoma*	NC_046704_1	Naticoidea	Naticidae	15,235 bp
*Tanea lineata*	NC_050662_1	Naticoidea	Naticidae	15,156 bp
*Naticarius hebraeus*	NC_028002_1	Naticoidea	Naticidae	15,384 bp
*Paratectonatica tigrina*	NC_050661_1	Naticoidea	Naticidae	15,201 bp
*Euspira gilva*	NC_046593_1	Naticoidea	Naticidae	15,315 bp
*Euspira pila*	NC_046703_1	Naticoidea	Naticidae	15,244 bp
*Glossaulax reiniana*	NC_041162_1	Naticoidea	Naticidae	15,254 bp
*Neverita didyma*	NC_046594_1	Naticoidea	Naticidae	15,629 bp
*Neverita* sp. *HL 2020*	MK500870_1	Naticoidea	Naticidae	15,190 bp
*Xenophora japonica*	NC_059926_1	Xenophoroidea	Xenophoridae	15,684 bp
*Struthiolaria papulosa*	NC_059921_1	Stromboidea	Struthiolaridae	15,475 bp
*Aporrhais serresiana*	NC_059920_1	Stromboidea	Aporrhaidae	15,455 bp
*Terebellum terebellum*	NC_059924_1	Stromboidea	Seraphsidae	15,478 bp
*Varicospira cancellata*	NC_059925_1	Stromboidea	Rostellariidae	15,864 bp
*Tibia fusus*	NC_065371_1	Stromboidea	Rostellariidae	16,083 bp
*Lentigo lentiginosus*	ON310809_1	Stromboidea	Strombidae	16,054 bp
*Euprotomus aratrum*	NC_084214_1	Stromboidea	Strombidae	16,187 bp
*Strombus pugilis*	NC_059922_1	Stromboidea	Strombidae	15,809 bp
*Aliger gigas*	MZ157283_1	Stromboidea	Strombidae	15,460 bp
	NC_024932_1	Stromboidea	Strombidae	15,461 bp
*Conomurex luhuanus*	NC_035726_1	Stromboidea	Strombidae	15,799 bp
*Laevistrombus canarium*	NC_053786_1	Stromboidea	Strombidae	15,626 bp
*D. vittatus*	PV623688	Stromboidea	Strombidae	16,239 bp
*Tridentarius dentatus*	NC_059923_1	Stromboidea	Strombidae	15,500 bp
*Canarium labiatum*	ON310803_1	Stromboidea	Strombidae	15,843 bp
*Harpago chiragra*	MH122656_1	Stromboidea	Strombidae	15,460 bp
*Lambis lambis*	MH115428_1	Stromboidea	Strombidae	15,481 bp
	NC_071230_1	Stromboidea	Strombidae	16,042 bp
	ON840106_1	Stromboidea	Strombidae	16,045 bp
*Volva habei*	NC_084101_1	Cypraeoidea	Ovulidae	16,519 bp
*Monetaria moneta*	NC_072229_1	Cypraeoidea	Cypraeidae	16,214 bp
*Monetaria caputserpentis*	NC_072232_1	Cypraeoidea	Cypraeidae	15,818 bp
*Naria helvola*	NC_072230_1	Cypraeoidea	Cypraeidae	15,648 bp
*Naria miliaris*	NC_072233_1	Cypraeoidea	Cypraeidae	16,123 bp
*Naria erosa*	NC_072231_1	Cypraeoidea	Cypraeidae	16,020 bp
*Lyncina vitellus*	NC_072226_1	Cypraeoidea	Cypraeidae	16,269 bp
*Cypraea tigris*	MK783263_1	Cypraeoidea	Cypraeidae	16,177 bp
*Mauritia arabica*	NC_072227_1	Cypraeoidea	Cypraeidae	15,855 bp
*Mauritia arabica asiatica*	MZ667219_1	Cypraeoidea	Cypraeidae	16,926 bp
*Purpuradusta gracilis*	NC_072228_1	Cypraeoidea	Cypraeidae	16,240 bp
*Erronea onyx*	NC_072234_1	Cypraeoidea	Cypraeidae	15,789 bp
*Erronea errones*	NC_066082_1	Cypraeoidea	Cypraeidae	15,422 bp
*Erronea caurica*	NC_072235_1	Cypraeoidea	Cypraeidae	15,857 bp
*Desmaulus extinctorium*	NC_079658_1	Calyptraeoidea	Calyptraeidae	16,608 bp
*Capulus dilatatus*	NC_084349_1	Capuloidea	Capulidae	15,640 bp
*Ficus variegata Roding*	NC_056153_1	Tonnoidea	Ficidae	15,736 bp
*Ficus subintermedia*	OR522697_1	Tonnoidea	Ficidae	16,255 bp
*Monoplex parthenopeus*	NC_013247_1	Tonnoidea	Ranellidae	15,270 bp
*T. chinensis*	PV623689	Tonnoidea	Tonnidae	16,241 bp
*Tonna galea*	NC_082277_1	Tonnoidea	Tonnidae	17,504 bp
*Tutufa rubeta*	NC_059877_1	Tonnoidea	Bursidae	15,397 bp
*Bursa rhodostoma*	MW316792_1	Tonnoidea	Bursidae	15,392 bp
	NC_054277_1	Tonnoidea	Bursidae	15,510 bp
	NC_059878_1	Tonnoidea	Bursidae	15,393 bp
*Galeodea echinophora*	NC_028003_1	Tonnoidea	Cassidae	15,388 bp
*P. glaucum*	PV623690	Tonnoidea	Cassidae	16,280 bp
*Charonia tritonis*	MT043269_1	Tonnoidea	Ranellidae	15,346 bp
	NC_082220_1	Tonnoidea	Ranellidae	15,346 bp
*Charonia lampas*	NC_037188_1	Tonnoidea	Ranellidae	15,405 bp
	KU237290_1	Tonnoidea	Ranellidae	15,330 bp

**Table 4:** General features of the complete mitochondrial genomes of *D. vittatus*, *T. chinensis*, and *P. glaucum*.

Species	*D. vittatus*	*T. chinensis*	*P. glaucum*
Genome size/bp	16,239	16,241	16,280
GC content/%	32.83	28.48	29.21
A%	29.52	31.96	32.21
T%	37.64	39.56	38.57
C%	16.99	13.06	14.21
G%	15.84	15.42	15.01
GC skew	0.035	0.062	0.027
AT skew	−0.121	−0.106	−0.090
rRNA/tRNA/CDS content	2/22/13	2/22/13	2/22/13
Protein-coding/%	69.35	69.27	69.08
Gene total length/bp	11,262	11,250	11,247

**Table 5:** Summary of the gene features of *D. vittatus*, *T. chinensis*, and *P. glaucum*.

Gene	Strand	Size/bp	Start_Codon	Stop_Codon
*cox3*	+	780 ^a,b,c^	ATG ^a,b,c^	TAA ^a,b,c^
*trnK*	+	70 ^a^/71 ^b^/69 ^c^		
*trnA*	+	71 ^a^/68 ^b,c^		
*trnR*	+	69 ^a,b,c^		
*trnN*	+	66 ^a^/68 ^b^/71 ^c^		
*trnI*	+	67 ^a^/71 ^b^/70 ^c^		
*nad3*	+	354 ^a,b,c^	ATG ^a,b,c^	TAA ^a,b,c^
*trnS1*	+	68 ^a,b,c^		
*nad2*	+	1059 ^a,b,c^	ATG ^a,b,c^	TAA ^a,b,c^
*cox1*	+	1536 ^a,b,c^	ATG ^a,b,c^	TAG ^a^/TAA ^b,c^
*cox2*	+	687 ^a,b,c^	ATG ^a,b,c^	TAA ^a,b,c^
*trnD*	+	68 ^a,b,c^		
*atp8*	+	159 ^a,b,c^	ATG ^a,b,c^	TAA ^a,b,c^
*atp6*	+	696 ^a,b,c^	ATG ^a,b,c^	TAG ^a^/TAA ^b,c^
*trnM*	−	67 ^a,c^/68 ^b^		
*trnY*	−	66 ^a,b^/67 ^c^		
*trnC*	−	66 ^a,b^/64 ^c^		
*trnW*	−	67 ^a^/68 ^b^/66 ^c^		
*trnQ*	−	65 ^a,b,c^		
*trnG*	−	67 ^a,b^/68 ^c^		
*trnE*	-	68 ^a^/71 ^b^/72 ^c^		
*rrnS*	+	947 ^a^/887 ^b^/894 ^c^		
*trnV*	+	68 ^a,b^/67 ^c^		
*rrnL*	+	1373 ^a^/1352 ^b^/1372 ^c^		
*trnL1*	+	69 ^a,b,c^		
*trnL2*	+	69 ^a,b,c^		
*nad1*	+	942 ^a,b,c^	ATG ^a,b,c^	TAG ^a^/TAA ^b,c^
*trnP*	+	68 ^a^/69 ^b,c^		
*nad6*	+	510 ^a^/501 ^b,c^	ATT ^a^/ATG ^b,c^	TAA ^a,c^/TAG ^b^
*cob*	+	1140 ^a,b,c^	ATG ^a,b,c^	TAA ^a,b,c^
*trnS2*	+	66 ^a^/65 ^b^/64 ^c^		
*trnT*	-	67 ^a,b^/66 ^c^		
*nad4l*	+	297 ^a,b,c^	ATG ^a,b,c^	TAG ^a,b,c^
*nad4*	+	1374 ^a,c^/1377 ^b^	GTG ^a^/ATG ^b,c^	TAA ^a,b^/TAG ^c^
*trnH*	+	65 ^a,c^/66 ^b^		
*nad5*	+	1728 ^a^/1722 ^b,c^	ATG ^a,b,c^	TAG ^a^/TAA ^b,c^
*trnF*	+	69 ^a,b^/68 ^c^		

^a^ D. vittatus, ^b^ T. chinensis, ^c^ P. glaucum.

## Data Availability

Data will be made available upon request.

## Supplementary Materials

The following supporting information can be downloaded at: https://www.mdpi.com/article/10.3390/ani16142248/s1, Figure S1: Phylogenetic tree of Littorinimorpha based on sequences of 13 PCGs. Bayesian posterior probabilities are shown for each node. Table S1: *D. vittatus*.tRNA.vs OR995294 tRNA blastn comparison results. Table S2: *T. chinensis*.tRNA.vs OR995294 tRNA blastn comparison results. Table S3: *P. glaucum*.tRNA.vs OR995294 tRNA blastn comparison results. Table S4: Source and status of mitochondrial genomes used in this study. Table S5: Genomic base composition. Table S6: Relative Synonymous Codon Usage (RSCU) values in the mitochondrial protein-coding genes of *T. chinensis*, *D. vittatus*, and *P. glaucum*. References [63,64,65,66,67] are cited in the Supplementary Materials.
