# Supplementary material for: Characteristics of Mitochondrial Genomes and Phylogenetic Analysis of Three Species of Littorinimorpha Snails
Source: Animals (Basel). 2026 Jul 20;16(14):2248. doi: 10.3390/ani16142248 (PMC13405869; doi:10.3390/ani16142248)
Supplement: Supplementary file 1 [file animals-16-02248-s001.zip › animals-3815858-supplementary.pdf]

Supporting Information

Characteristics of mitochondrial genomes and phylogenetic analysis of three species of Littorinimorpha snails

Xumin Wang †, Minglei Li †, Xiaofei Lu, Chengen Tu, Fuyang He, Sutao Li, Dongyue Gu, Tianyi Liu, Pengyu Qu, Zhikai Xing, Shuang Wang, Lijun Wang * and Jiangyong Qu *

College of Life Science, Yantai University, Yantai 264005, China
* Correspondence: wanglijun@ytu.edu.cn (L.W.); qjy@ytu.edu.cn (J.Q.)
† These authors contributed equally to this work.

This file contains supplementary 1 Figure and 5 tables:

Figure S1. Phylogenetic tree of Littorinimorpha based on sequences of 13 PCGs. Bayesian posterior probabilities are shown for each node.

Table S1. *D. vittatus*.tRNA.vs OR995294 tRNA blastn comparison results.

Table S2. *T. chinensis*.tRNA.vs OR995294 tRNA blastn comparison results.

Table S3. *P. glaucum*.tRNA.vs OR995294 tRNA blastn comparison results.

Table S4. Source and status of mitochondrial genomes used in this study.

Table S5. Genomic base composition.

Table S6. Relative Synonymous Codon Usage (RSCU) values in the mitochondrial protein-coding genes of *T. chinensis*, *D. vittatus*, and *P. glaucum*.

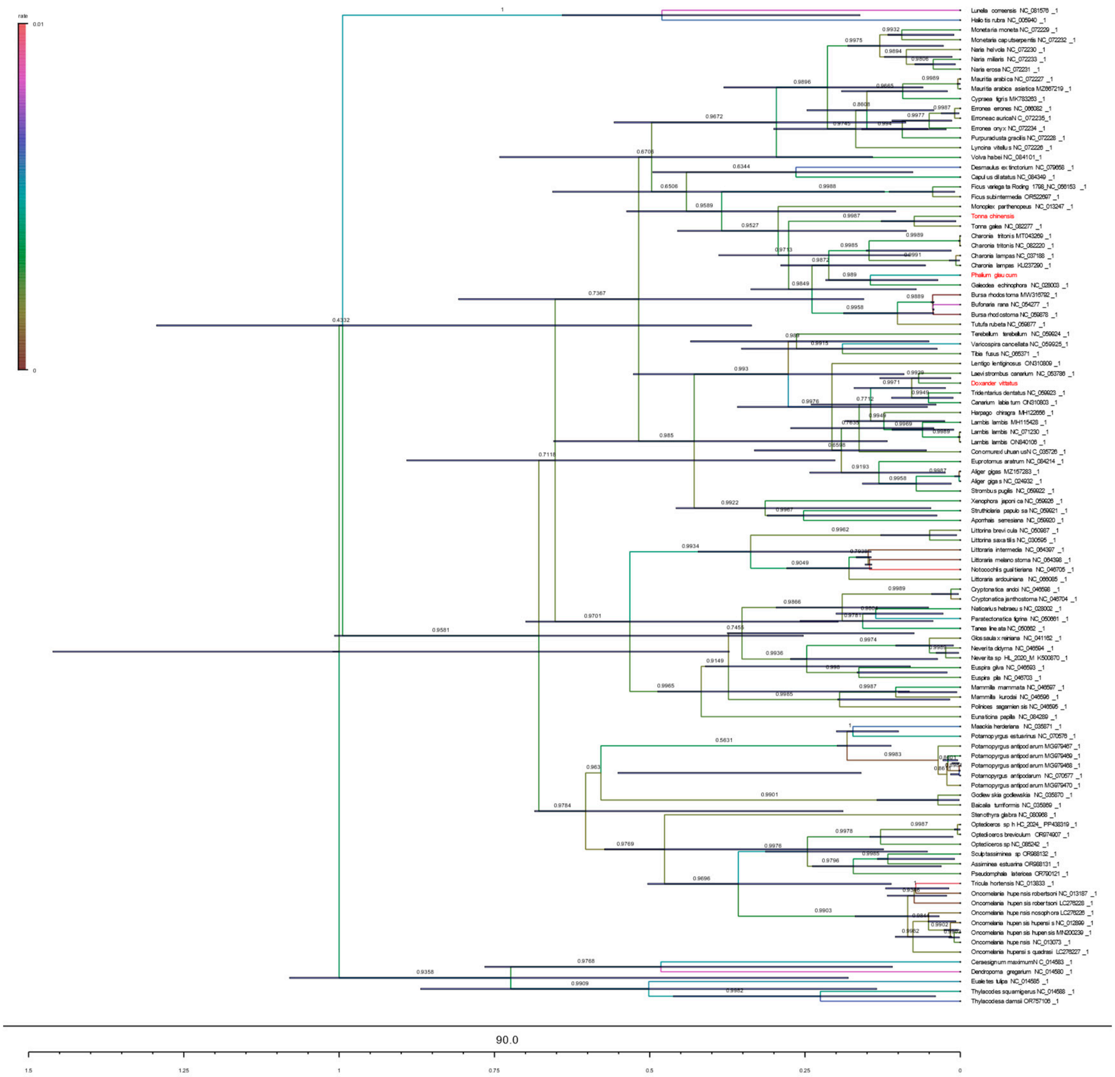

Figure S1. Phylogenetic tree of Littorinimorpha based on sequences of 13 PCGs. Bayesian posterior probabilities are shown for each node.

Table S1. *D. vittatus*.tRNA.vs OR995294 tRNA blastn comparison results

Query_id	Query length	Query start	Query end	Subject id	Subject length	Subject start	Subject end	Identity	Gap	Align length	Score	E_value	Subject annotation
tRNA-Lys	70	5	70	trnK(ttt)	70	5	70	0.93	0	66	102	1e-26	813-882;+;
tRNA-Ala	71	1	71	trnA(tgc)	71	1	71	0.98	0	71	123	4e-33	909-979;+;
tRNA-Arg	69	1	69	trnR(tcg)	69	1	69	0.95	0	69	111	2e-29	988-1056;+;
tRNA-Asn	68	1	68	trnN(gtt)	66	1	66	0.94	0.02	68	98.7	1e-25	1069-1134;+;
tRNA-Ile	67	1	67	trnI(gat)	67	1	67	0.94	0	67	104	3e-27	1136-1202;+;
tRNA-Ser	68	1	68	trnS1(gct)	68	1	68	1	0	68	123	4e-33	1560-1627;+;
tRNA-Asp	68	1	68	trnD(gtc)	68	1	68	1	0	68	123	4e-33	4932-4999;+;
tRNA-Met	67	1	67	trnM(cat)	67	1	67	0.97	0	67	113	6e-30	5898-5964;-;
tRNA-Tyr	66	1	64	trnY(gta)	66	1	64	0.96	0	64	107	3e-28	5982-6047;-;
tRNA-Cys	66	1	66	trnC(gca)	66	1	66	0.93	0	66	102	1e-26	6050-6115;-;
tRNA-Trp	67	1	67	trnW(tca)	67	1	67	0.98	0	67	116	5e-31	6117-6183;-;
tRNA-Gln	62	1	62	trnQ(ttg)	65	1	62	1	0	62	113	6e-30	6182-6246;-;
tRNA-Gly	67	1	67	trnG(tcc)	67	1	67	0.97	0	67	113	6e-30	6257-6323;-;
tRNA-Glu	70	1	70	trnE(ttc)	68	1	68	0.92	0.02	70	96.9	5e-25	6325-6392;-;
tRNA-Val	69	1	69	trnV(tac)	68	1	68	0.95	0.01	69	107	3e-28	7368-7435;+;
tRNA-Leu	69	1	69	trnL1(tag)	69	1	69	0.98	0	69	120	4e-32	8809-8877;+;
tRNA-Leu	69	1	69	trnL2(taa)	69	1	69	1	0	69	125	1e-33	8884-8952;+;
tRNA-Pro	68	4	68	trnP(tgg)	68	4	68	0.95	0	65	104	3e-27	9908-9975;+;
tRNA-Ser	66	1	66	trnS2(tga)	66	1	66	0.96	0	66	111	2e-29	11642-11707;+;
tRNA-Thr	68	1	68	trnT(tgt)	67	1	67	0.95	0.01	68	105	9e-28	11716-11782;-;
tRNA-His	65	1	65	trnH(gtg)	65	1	65	0.98	0	65	113	6e-30	13457-13521;+;

Table S2. *T. chinensis*.tRNA.vs OR995294 tRNA blastn comparison results

Query_id	Query length	Query start	Query end	Subject id	Subject length	Subject start	Subject end	Identity	Gap	Align length	Score	E_value	Subject annotation
tRNA-Ala	71	1	55	trnA(tgc)	68	1	55	0.85	0	55	64.4	3e-15	875-942;+;
tRNA-Arg	69	5	67	trnR(tcg)	69	5	67	0.88	0	63	82.4	1e-20	963-1031;+;
tRNA-Asn	68	1	68	trnN(gtt)	68	1	68	0.85	0	68	78.8	1e-19	1036-1103;+;
tRNA-Ile	67	6	49	trnI(gat)	71	6	49	0.97	0	44	75.2	2e-18	1129-1199;+;
tRNA-Ser	68	1	68	trnS1(gct)	68	1	68	1	0	68	123	4e-33	1563-1630;+;
tRNA-Asp	68	1	68	trnD(gtc)	68	1	68	0.92	0	68	100	4e-26	4941-5008;+;
tRNA-Met	67	1	67	trnM(cat)	68	1	68	0.91	0.01	68	93.3	6e-24	5930-5997;-;
tRNA-Tyr	66	1	66	trnY(gta)	66	1	66	0.87	0	66	84.2	3e-21	6003-6068;-;
tRNA-Cys	66	1	48	trnC(gca)	66	1	49	0.81	0.02	49	44.6	3e-09	6078-6143;-;
tRNA-Trp	67	1	67	trnW(tca)	68	1	68	0.88	0.01	68	84.2	3e-21	6144-6211;-;
tRNA-Gln	62	1	62	trnQ(ttg)	65	1	62	0.87	0	62	77	4e-19	6211-6275;-;
tRNA-Gly	67	3	60	trnG(tcc)	67	3	60	0.86	0	58	69.8	7e-17	6278-6344;-;
tRNA-Glu	70	1	70	trnE(ttc)	71	1	71	0.84	0.01	71	75.2	2e-18	6345-6415;-;
tRNA-Val	69	1	69	trnV(tac)	68	1	68	0.73	0.01	69	41	3e-08	7379-7446;+;
tRNA-Leu	69	3	69	trnL1(tag)	69	3	69	0.88	0	67	86	9e-22	8842-8910;+;
tRNA-Leu	69	1	69	trnL2(taa)	69	1	69	0.89	0	69	93.3	6e-24	8912-8980;+;
tRNA-Pro	68	4	68	trnP(tgg)	69	4	69	0.77	0.01	66	48.2	2e-10	9923-9991;+;
tRNA-Ser	66	8	57	trnS2(tga)	65	8	56	0.86	0.02	50	55.4	1e-12	11648-11712;+;
tRNA-Thr	68	1	68	trnT(tgt)	67	1	67	0.81	0.07	70	50	6e-11	11717-11783;-;
tRNA-His	65	4	51	trnH(gtg)	66	4	53	0.78	0.04	50	33.7	5e-06	13472-13537;+;
tRNA-Phe	70	7	53	trnF(gaa)	69	7	54	0.81	0.02	48	42.8	1e-08	15260-15328;+;
tRNA-Ala	71	1	55	trnA(tgc)	68	1	55	0.85	0	55	64.4	3e-15	875-942;+;

Table S3. *P. glaucum*.tRNA.vs OR995294 tRNA blastn comparison results

Query_id	Query length	Query start	Query end	Subject id	Subject length	Subject start	Subject end	Identity	Gap	Align length	Score	E_value	Subject annotation
tRNA-Ala	71	1	71	trnA(tgc)	68	1	68	0.83	0.06	72	71.6	2e-17	878-945;+;
tRNA-Arg	69	5	60	trnR(tcg)	69	5	60	0.89	0	56	75.2	2e-18	952-1020;+;
tRNA-Asn	68	1	68	trnN(gtt)	71	1	71	0.81	0.06	72	68	2e-16	1035-1105;+;
tRNA-Ile	67	6	61	trnI(gat)	70	6	64	0.93	0.05	59	82.4	1e-20	1107-1176;+;
tRNA-Ser	68	1	68	trnS1(gct)	68	1	68	1	0	68	123	4e-33	1539-1606;+;
tRNA-Asp	68	1	68	trnD(gtc)	68	1	68	0.97	0	68	114	2e-30	4916-4983;+;
tRNA-Met	67	1	67	trnM(cat)	67	1	67	0.89	0.02	68	84.2	3e-21	5898-5964;-;
tRNA-Tyr	66	1	66	trnY(gta)	67	1	67	0.88	0.01	67	82.4	1e-20	5982-6048;-;
tRNA-Cys	66	1	66	trnC(gca)	64	1	64	0.8	0.03	66	53.6	5e-12	6059-6122;-;
tRNA-Trp	67	1	67	trnW(tca)	66	1	66	0.85	0.01	67	73.4	6e-18	6123-6188;-;
tRNA-Gln	62	1	62	trnQ(ttg)	65	1	62	0.95	0	62	98.7	1e-25	6187-6251;-;
tRNA-Gly	67	3	60	trnG(tcc)	68	3	61	0.88	0.01	59	71.6	2e-17	6257-6324;-;
tRNA-Glu	70	21	70	trnE(ttc)	72	23	72	0.92	0	50	73.4	6e-18	6331-6402;-;
tRNA-Val	69	1	69	trnV(tac)	67	1	67	0.75	0.02	69	41	3e-08	7369-7435;+;
tRNA-Leu	69	4	65	trnL1(tag)	69	4	65	0.93	0	62	95.1	2e-24	8847-8915;+;
tRNA-Leu	69	1	69	trnL2(taa)	69	1	69	0.89	0	69	93.3	6e-24	8916-8984;+;
tRNA-Pro	68	4	68	trnP(tgg)	69	4	69	0.81	0.01	66	62.6	1e-14	9927-9995;+;
tRNA-Ser	66	7	66	trnS2(tga)	64	6	64	0.81	0.01	60	55.4	1e-12	11660-11723;+;
tRNA-Thr	68	1	68	trnT(tgt)	66	1	66	0.83	0.02	68	66.2	8e-16	11724-11789;-;
tRNA-His	65	4	61	trnH(gtg)	65	4	61	0.76	0.03	59	37.4	4e-07	13476-13540;+;
tRNA-Ala	71	1	71	trnA(tgc)	68	1	68	0.83	0.06	72	71.6	2e-17	878-945;+;
tRNA-Arg	69	5	60	trnR(tcg)	69	5	60	0.89	0	56	75.2	2e-18	952-1020;+;

Table S4. Source and status of mitochondrial genomes used in this study

Species	NCBI accession	Total reads	Status	Reference
Lunella correensis	NC_081576_1	17,309 bp	Unpublished	BioProject: PRJNA927338
Haliotis rubra	NC_005940_1	16,907 bp	Published	[S1]
Ceraesignum maximum	NC_014583_1	15,578 bp	Published	[S2]
Dendropoma gregarium	NC_014580_1	15,641 bp	Published	[S2]
Eualetes tulipa	NC_014585_1	15,078 bp	Published	[S2]
Thylacodes squamigerus	NC_014588_1	15,544 bp	Published	[S2]
Thylacodes adamsii	OR757106_1	14,913 bp	Published	[S3]
Maackia herderiana	NC_035871_1	15,154 bp	Unpublished	BioProject: PRJNA927338
Godlewskia godlewskia	NC_035870_1	15,224 bp	Unpublished	BioProject: PRJNA927338
Baicalia turriformis	NC_035869_1	15,127 bp	Unpublished	BioProject: PRJNA927338
Potamopyrgus estuarinus	NC_070576_1	16,701 bp	Published	[S4]
Potamopyrgus antipodarum	MG979467_1	15,141 bp	Published	[S5]
	NC_070577_1	16,846 bp	Published	[S4]
	MG979470_1	15,149 bp	Published	[S5]
	MG979469_1	15,145 bp	Published	[S5]
	MG979468_1	15,149 bp	Published	[S5]
Stenothyra glabra	NC_080968_1	15,704 bp	Unpublished	BioProject: PRJNA927338
Optediceros sp	NC_085242_1	15,906 bp	Unpublished	BioProject: PRJNA927338
Optediceros sp. h HC 2024	PP438319_1	15,969 bp	Unpublished	Submitted by: Hc, C.D, 2024
Optediceros breviculum	OR974907_1	15,870 bp	Unpublished	Submitted by: Hc, C.D, 2023
Pseudomphala latericea	OR790121_1	16,635 bp	Unpublished	Submitted by: Yuan,Y, 2023
Sculptassiminea sp.	OR988132_1	16,073 bp	Unpublished	Submitted by: Hc, C.D, 2023
Assiminea estuarina	OR988131_1	15,907 bp	Unpublished	Submitted by: Hc, C.D, 2023
Tricula hortensis	NC_013833_1	15,179 bp	Unpublished	BioProject: PRJNA927338
Oncomelania hupensis robertsoni	LC276228_1	15,188 bp	Unpublished	Submitted by: Hino,A, 2017
	NC_013187_1	15,191 bp	Unpublished	BioProject: PRJNA927338
Oncomelania hupensis quadrasi	LC276227_1	15,184 bp	Unpublished	Submitted by: Hino,A, 2017
Oncomelania hupensis nosophora	LC276226_1	15,182 bp	Unpublished	Submitted by: Hino,A, 2017
Oncomelania hupensis hupensis	NC_012899_1	15,186 bp	Unpublished	Submitted by: Zhzo,Q.P, 2007
	NC_013073_1	15,182 bp	Unpublished	Submitted by: Li,S, 2009
	MN200239_1	15,850 bp	Unpublished	Submitted by: Mao,Q, 2019
Littorina brevicula	NC_050987_1	16,356 bp	Unpublished	Submitted by: Bai,J, 2020
Littorina saxatilis	NC_030595_1	16,887 bp	Unpublished	BioProject: PRJNA927338
Littoraria ardouiniana	NC_066085_1	16,261 bp	Unpublished	BioProject: PRJNA927338
Littoraria intermedia	NC_064397_1	16,194 bp	Unpublished	BioProject: PRJNA927338
Littoraria melanostoma	NC_064398_1	16,149 bp	Unpublished	BioProject: PRJNA927338
Notocochlis gualtieriana	NC_046705_1	15,176 bp	Unpublished	BioProject: PRJNA927338
Eunaticina papilla	NC_084289_1	16,415 bp	Unpublished	BioProject: PRJNA927338
Polinices sagamiensis	NC_046595_1	15,383 bp	Unpublished	BioProject: PRJNA927338
Mammilla mammata	NC_046597_1	15,319 bp	Unpublished	BioProject: PRJNA927338

Mammilla kurodai	NC_046596_1	15,309 bp	Unpublished	BioProject: PRJNA927338
Cryptonatica andoi	NC_046598_1	15,302 bp	Unpublished	BioProject: PRJNA927338
Cryptonatica janthostoma	NC_046704_1	15,235 bp	Unpublished	BioProject: PRJNA927338
Tanea lineata	NC_050662_1	15,156 bp	Unpublished	BioProject: PRJNA927338
Naticarius hebraeus	NC_028002_1	15,384 bp	Published	[S6]
Paratectonatica tigrina	NC_050661_1	15,201 bp	Unpublished	BioProject: PRJNA927338
Euspira gilva	NC_046593_1	15,315 bp	Unpublished	BioProject: PRJNA927338
Euspira pila	NC_046703_1	15,244 bp	Unpublished	BioProject: PRJNA927338
Glossaulax reiniana	NC_041162_1	15,254 bp	Unpublished	BioProject: PRJNA927338
Neverita didyma	NC_046594_1	15,629 bp	Unpublished	BioProject: PRJNA927338
Neverita sp.HL 2020	MK500870_1	15,190 bp	Unpublished	Submitted by: Liu,h, 2020
Xenophora japonica	NC_059926_1	15,684 bp	Published	[S7]
Struthiolaria papulosa	NC_059921_1	15,475 bp	Published	[S7]
Aporrhais serresiana	NC_059920_1	15,455 bp	Published	[S7]
Terebellum terebellum	NC_059924_1	15,478 bp	Published	[S7]
Varicospira cancellata	NC_059925_1	15,864 bp	Published	[S7]
Tibia fusus	NC_065371_1	16,083 bp	Unpublished	BioProject: PRJNA927338
Lentigo lentiginosus	ON310809_1	16,054 bp	Unpublished	Submitted by: Li,F, 2022
Euprotomus aratrum	NC_084214_1	16,187 bp	Unpublished	BioProject: PRJNA927338
Strombus pugilis	NC_059922_1	15,809 bp	Published	[S7]
Aliger gigas	MZ157283_1	15,460 bp	Unpublished	Submitted by: Machkour-M'Rabet,S, 2021
	NC_024932_1	15,461 bp	Unpublished	Submitted by: Machkour-M'Rabet,S, 2014
Conomurex luhuanus	NC_035726_1	15,799 bp	Unpublished	BioProject: PRJNA927338
Laevistrombus canarium	NC_053786_1	15,626 bp	Unpublished	BioProject: PRJNA927338
D. vittatus	PV623688	16,239 bp	Unpublished	This study
Tridentarius dentatus	NC_059923_1	15,500 bp	Published	[S7]
Canarium labiatum	ON310803_1	15,843 bp	Unpublished	Submitted by: Li,F, 2022
Harpago chiragra	MH122656_1	15,460 bp	Published	[S8]
	MH115428_1	15,481 bp	Published	[S8]
Lambis lambis	NC_071230_1	16,042 bp	Unpublished	BioProject: PRJNA927338
	ON840106_1	16,045 bp	Unpublished	Submitted by: Li,F, 2022
Volva habei	NC_084101_1	16,519 bp	Unpublished	BioProject: PRJNA927338
Monetaria moneta	NC_072229_1	16,214 bp	Unpublished	BioProject: PRJNA927338
Monetaria caputserpentis	NC_072232_1	15,818 bp	Unpublished	BioProject: PRJNA927338
Naria helvola	NC_072230_1	15,648 bp	Unpublished	BioProject: PRJNA927338
Naria miliaris	NC_072233_1	16,123 bp	Unpublished	BioProject: PRJNA927338
Naria erosa	NC_072231_1	16,020 bp	Unpublished	BioProject: PRJNA927338
Lyncina vitellus	NC_072226_1	16,269 bp	Unpublished	BioProject: PRJNA927338
Cypraea tigris	MK783263_1	16,177 bp	Unpublished	Submitted by: Liu,H, 2019
Mauritia arabica	NC_072227_1	15,855 bp	Unpublished	BioProject: PRJNA927338
Mauritia arabica asiatica	MZ667219_1	16,926 bp	Unpublished	Submitted by: Qu,J, 2021
Purpuradusta gracilis	NC_072228_1	16,240 bp	Unpublished	BioProject: PRJNA927338
Erronea onyx	NC_072234_1	15,789 bp	Unpublished	BioProject: PRJNA927338
Erronea errones	NC_066082_1	15,422 bp	Unpublished	BioProject: PRJNA927338

Erronea caurica	NC_072235_1	15,857 bp	Unpublished	BioProject: PRJNA927338
Desmaulus extinctorium	NC_079658_1	16,608 bp	Unpublished	BioProject: PRJNA927338
Capulus dilatatus	NC_084349_1	15,640 bp	Unpublished	BioProject: PRJNA927338
Ficus variegata Roding	NC_056153_1	15,736 bp	Unpublished	BioProject: PRJNA927338
Ficus subintermedia	OR522697_1	16,255 bp	Unpublished	Submitted by: Ma,Y, 2023
Monoplex parthenopeus	NC_013247_1	15,270 bp	Published	[S9]
T. chinensis	PV623689	16,241 bp	Unpublished	This study
Tonna galea	NC_082277_1	17,504 bp	Unpublished	BioProject: PRJNA927338
Tutufa rubeta	NC_059877_1	15,397 bp	Unpublished	BioProject: PRJNA927338
	MW316792_1	15,392 bp	Unpublished	Submitted by: Sanders,M.T.2020
Bursa rhodostoma	NC_054277_1	15,510 bp	Unpublished	BioProject: PRJNA927338
	NC_059878_1	15,393 bp	Unpublished	BioProject: PRJNA927338
Galeodea echinophora	NC_028003_1	15,388 bp	Published	[S6]
P. glaucum	PV623690	16,280 bp	Unpublished	This study
Charonia tritonis	MT043269_1	15,346 bp	Published	Submitted by: Zhang,X, 2020
	NC_082220_1	15,346 bp	Unpublished	BioProject: PRJNA927338
Charonia lampas	NC_037188_1	15,405 bp	Unpublished	BioProject: PRJNA927338
	KU237290_1	15,330 bp	Published	[S10]

(S1) Maynard, B. T.; Kerr, L. J.; McKiernan, J. M.; Jansen, E. S.; Hanna, P. J. Mitochondrial DNA sequence and gene organization in the [corrected] Australian blacklip [corrected] abalone Haliotis rubra (leach). *Mar Biotechnol (NY)* **2005**, *7* (6), 645-658. DOI: 10.1007/s10126-005-0013-z.

(S2) Rawlings, T. A.; MacInnis, M. J.; Bieler, R.; Boore, J. L.; Collins, T. M. Sessile snails, dynamic genomes: gene rearrangements within the mitochondrial genome of a family of caenogastropod molluscs. *BMC Genomics* **2010**, *11*, 440. DOI: 10.1186/1471-2164-11-440.

(S3) Lee, Y.; Kim, K. B.; Choi, E. H.; Hwang, U. W. Complete mitochondrial genome of the worm snail Thylacodes adamsii (Littorinimorpha: Vermetidae) from South Korea. *Mitochondrial DNA B Resour* **2024**, *9* (6), 753-757. DOI: 10.1080/23802359.2024.2368209.

(S4) Sharbrough, J.; Bankers, L.; Cook, E.; Fields, P. D.; Jalinsky, J.; McElroy, K. E.; Neiman, M.; Logsdon, J. M.; Boore, J. L. Single-molecule Sequencing of an Animal Mitochondrial Genome Reveals Chloroplast-like Architecture and Repeat-mediated Recombination. *Mol Biol Evol* **2023**, *40* (1), 1-13. DOI: 10.1093/molbev/msad007.

(S5) Sharbrough, J.; Luse, M.; Boore, J. L.; Logsdon, J. M., Jr.; Neiman, M. Radical amino acid mutations persist longer in the absence of sex. *Evolution* **2018**, *72* (4), 808-824. DOI: 10.1111/evo.13465.

(S6) Osca, D.; Templado, J.; Zardoya, R. Caenogastropod mitogenomics. *Mol Phylogenet Evol* **2015**, *93*, 118-128. DOI: 10.1016/j.ympev.2015.07.011.

(S7) Irwin, A. R.; Strong, E. E.; Kano, Y.; Harper, E. M.; Williams, S. T. Eight new mitogenomes clarify the phylogenetic relationships of Stromboidea within the caenogastropod phylogenetic framework. *Mol Phylogenet Evol* **2021**, *158*, 107081. DOI: 10.1016/j.ympev.2021.107081.

(S8) Jiang, D.; Zheng, X.; Zeng, X.; Kong, L.; Li, Q. The complete mitochondrial genome of Harpago chiragra and Lambis lambis (Gastropoda: Stromboidea): implications on the Littorinimorpha phylogeny. *Sci Rep* **2019**, *9* (1), 17683. DOI: 10.1038/s41598-019-54141-x.

(S9) Cunha, R. L.; Grande, C.; Zardoya, R. Neogastropod phylogenetic relationships based on entire mitochondrial genomes. *BMC Evol Biol* **2009**, *9*, 210. DOI: 10.1186/1471-2148-9-210.

(S10) Choi, E. H.; Hwang, U. W. The complete mitochondrial genome of an endangered triton snail Charonia lampas (Littorinimorpha: Charoniidae) from South Korea. *Mitochondrial DNA B Resour* **2021**, *6* (3), 956-958. DOI: 10.1080/23802359.2021.1889416.

Table S5. Genomic base composition

Species	Region	Length(bp)	T%	C%	A%	G%	AT%	GC%
D. vittatus	Genome	16,239	37.64	15.84	29.52	16.99	67.17	32.83
	PCGs	11,262	39.53	16.45	27.13	16.89	66.66	33.34
	tRNA	1,486	31.76	14.47	34.25	19.52	66.02	33.98
	rRNA	2,320	32.67	13.23	36.47	17.63	69.14	30.86
T. chinensis	Genome	16,241	39.56	13.06	31.96	15.42	71.52	28.48
	PCGs	11,250	41.06	13.64	29.55	15.76	70.6	29.4
	tRNA	1,496	34.36	12.97	35.56	17.11	69.92	30.08
	rRNA	2,239	34.84	10.94	38.37	15.86	73.2	26.8
P. glaucum	Genome	16,280	38.57	14.21	32.21	15.01	70.78	29.22
	PCGs	11,247	40.14	15.05	29.7	15.11	69.84	30.16
	tRNA	1,489	34.45	12.36	36.27	16.92	70.72	29.28
	rRNA	2,266	33.58	11.69	38.83	15.89	72.42	27.58

Table S6. Relative Synonymous Codon Usage (RSCU) values in the mitochondrial protein-coding genes of *T. chinensis*, *D. vittatus*, and *P. glaucum*

**Codon**	**AA**	**RSCU**		
		T. chinensis	***D. vittatus***	***P. glaucum***
GCA	Ala	1.546	1	1.333
GCC	Ala	0.336	0.667	0.578
GCG	Ala	0.118	0.136	0.161
GCT	Ala	2	2.197	1.928
CGA	Arg	1.105	1.235	1.871
CGC	Arg	0.039	0.088	0.091
CGG	Arg	0.197	0.485	0.023
CGT	Arg	1.026	0.838	0.73
AAC	Asn	0.211	0.453	0.421
AAT	Asn	1.789	1.547	1.579
GAC	Asp	0.427	0.526	0.384
GAT	Asp	1.573	1.474	1.616
TGC	Cys	0.462	0.35	0.186
TGT	Cys	1.538	1.65	1.814
CAA	Gln	1.59	1.243	1.684
CAG	Gln	0.41	0.757	0.316
GAA	Glu	1.814	1.415	1.696
GAG	Glu	0.186	0.585	0.304
GGA	Gly	1.806	1.558	2.242
GGC	Gly	0.323	0.337	0.258
GGG	Gly	0.403	0.659	0.419
GGT	Gly	1.468	1.446	1.081
CAC	His	0.35	0.571	0.456

CAT	His	1.65	1.429	1.544
ATC	Ile	0.162	0.291	0.173
ATT	Ile	1.715	1.77	1.721
CTA	Leu1	0.71	0.882	0.742
CTC	Leu1	0.062	0.287	0.146
CTG	Leu1	0.134	0.185	0.052
CTT	Leu1	0.792	1.179	1.348
TTA	Leu2	3.664	2.892	3.418
TTG	Leu2	0.638	0.574	0.293
AAA	Lys	1.711	1.453	1.812
AAG	Lys	0.289	0.547	0.188
ATA	Met	1.123	0.938	1.106
ATG	Met	1	1	1
TTC	Phe	0.184	0.482	0.351
TTT	Phe	1.816	1.518	1.649
CCA	Pro	1.687	1.427	1.514
CCC	Pro	0.245	0.42	0.351
CCG	Pro	0.136	0.196	0.189
CCT	Pro	1.932	1.958	1.946
AGA	Ser1	3.039	2.912	2.738
AGC	Ser1	0.214	0.471	0.252
AGG	Ser1	0.592	0.441	0.548
AGT	Ser1	1.093	1	1.133
TCA	Ser2	1.779	1.49	1.93
TCC	Ser2	0.321	0.627	0.42
TCG	Ser2	0.3	0.176	0.189
TCT	Ser2	2.293	2.235	2.077
ACA	Thr	1.609	1.506	1.545
ACC	Thr	0.253	0.315	0.386
ACG	Thr	0.207	0.225	0.182
ACT	Thr	1.931	1.955	1.886
TGG	Trp	1	1	1
TAC	Tyr	0.196	0.507	0.4
TAT	Tyr	1.804	1.493	1.6
GTA	Val	1.714	1.542	1.768
GTC	Val	0.196	0.397	0.223
GTG	Val	0.359	0.458	0.24
GTT	Val	1.731	1.603	1.768

* AA: encoded amino acid
